# Effective Suckling C57BL/6, Kunming, and BALB/c Mouse Models with Remarkable Neurological Manifestation for Zika Virus Infection

**DOI:** 10.3390/v9070165

**Published:** 2017-06-29

**Authors:** Jianhai Yu, Xuling Liu, Changwen Ke, Qinghua Wu, Weizhi Lu, Zhiran Qin, Xiaoen He, Yujing Liu, Jieli Deng, Suiqi Xu, Ying Li, Li Zhu, Chengsong Wan, Qiwei Zhang, Weiwei Xiao, Qian Xie, Bao Zhang, Wei Zhao

**Affiliations:** 1 Guangdong Provincial Key Laboratory of Tropical Disease Research, School of Public Health, Southern Medical University, Guangzhou 510515, China; chienhai@163.com (J.Y.); maiblume@163.com (X.L.); wuqh@smu.edu.cn (Q.W.); pigiraffe@126.com (W.L.); baobaofei666666@outlook.com (Z.Q.); hexiaoen24@163.com (X.H.); liuyujing0729@outlook.com (Y.Liu); 15626436064@163.com (J.D.); ikius33@163.com (S.X.); ly3130090083@sina.com (Y.Li); zhuli89@126.com (L.Z.); gzwcs@smu.edu.cn (C.W.); zhangqw@smu.edu.cn (Q.Z.); xweiwei74@126.com (W.X.); xiyuxie89@126.com (Q.X.); 2 Institute of Microbiology, Center for Diseases Control and Prevention of Guangdong Province, 176 Xin Gang West Road, Guangzhou, Guangdong 510300, China; Kecw1965@aliyun.com; 3 Guangzhou Key Laboratory of Drug Research for Emerging Virus Prevention and Treatment, School of Pharmacy, Southern Medical University, Guangzhou 510515, China

**Keywords:** Zika virus, Guillain–Barré syndrome (GBS), suckling mouse model, C57BL/6 mice, Kunming mice, BALB/c mice, neurologic manifestation

## Abstract

Since 2015, 84 countries and territories reported evidence of vector-borne Zika Virus (ZIKV) transmission. The World Health Organization (WHO) declared that ZIKV and associated consequences especially the neurological autoimmune disorder Guillain–Barré syndrome (GBS) and microcephaly will remain a significant enduring public health challenge requiring intense action. We apply a standardization of the multi-subcutaneous dorsal inoculation method to systematically summarize clinical neurological manifestation, viral distribution, and tissue damage during the progress of viremia and systemic spread in suckling mouse models. We found that C57BL/6 and Kunming mice (KM) both showed remarkable and uniform neurologic manifestations. C57BL/6 owned the highest susceptibility and pathogenicity to the nervous system, referred to as movement disorders, with 100% incidence, while KM was an economic model for a Chinese study characterized by lower limb weakness with 62% morbidity. Slight yellow extraocular exudates were observed in BALB/c, suggesting the association with similar ocular findings to those of clinical cases. The virus distribution and pathological changes in the sera, brains, livers, kidneys, spleens, and testes during disease progression had strong regularity and uniformity, demonstrating the effectiveness and plasticity of the animal models. The successful establishment of these animal models will be conducive to expound the pathogenic mechanism of GBS.

## 1. Introduction

Zika virus (ZIKV) belongs to the *Flavivirus* genus of the *Flaviviridae* family, which contains many viruses that cause human diseases, such as dengue, yellow fever, Japanese encephalitis, and West Nile encephalitis [1]. After ZIKV was first isolated in Uganda in 1947 [2], ZIKV infection was regarded as asymptomatic [3], until outbreaks of febrile disease in the Yap Islands of the Federated States of Micronesia, French Polynesia, and Oceania occurred in 2007 [4]. After French Polynesia reported autochthonous cases of ZIKV infection in 2013, the virus began to spread rapidly [5]. In 2015, ZIKV was reported in Brazil, causing extensive concern owing to the number and severity of cases [6]. Recent studies have indicated that, in addition to mosquito transmission, ZIKV could spread through sexual contact, blood transmission, and mother-to-child transmission [7], in which a short period of viremia is followed by spread to various organs, leading to various complications, such as the neurological autoimmune disorder Guillain–Barré syndrome (GBS) and microcephaly in the infants of mothers infected during pregnancy [8,9]. As a result, the World Health Organization (WHO) declared that Zika virus and associated consequences still remain a significant enduring public health challenge requiring intense action [10]. According to the WHO, since 2015, 84 countries and territories reported evidence of vector-borne ZIKV transmission and more than 60 global and local partners are contributing to the ZIKV response [11].

Successful vaccines, medicines, or antivirals against ZIKV infection have not been developed [12,13,14]. Thus, it is necessary to establish an animal model to investigate the pathogenic mechanism and to develop treatment and prevention strategies. Several ZIKV animal models have been studied [15,16,17,18,19,20,21,22,23,24,25,26], but they have various limitations. Susceptible animal models for ZIKV infection or disease were first studied in the 1950s using rhesus macaques [2]. Mice deficient in the IFN-α/β receptors (A129) or IFN-α/β and Ɣ receptors (AG129) [16,17,18] are expensive and have a narrow range of applications owing to incomplete immunoregulatory mechanisms. Accordingly, a more economical and broadly applicable murine model is urgently needed. 

Kunming mice (KM), C57BL/6 mice (C57BL/6), and BALB/c mice (BALB/c) are regarded as potential viral infection animal models. KM are outbred, but exhibit minimal variation in growth and reproductive performance, and C57BL/6 and BALB/c are inbred strains. KM was first originated from Swiss mice, which is applied for toxicology and neurology studies [27,28]. Nowadays, KM is the most highly produced in China; it is used in pharmacology and toxicology studies owing to its high yield, good quality, resistance, and strong adaptability. Especially, for Chinese researchers, owing to its flexible neurological signs and symptoms, KM is useful in neurophysiology and neuroethology in the study of congenital human cytomegalovirus infection [29], dementia, anxiety neurosis, stroke, etc. [30,31]. Additionally, KM is also a usage model for central nervous system (CNS) research, like neural tube defects and apoptosis of cerebral nerve cells induced by stimulants. Considering the preferential homing of ZIKV to the CNS, KM is an important candidate for studying CNS damage caused by ZIKV infection. C57BL/6 is widely used in physiological and pathological research [32,33]. Huang et al. have reported that intracranial injections of ZIKV into C57BL/6 during maximal brain growth cause microcephaly and corticospinal neuron apoptosis [34]. Moreover, Manangeeswaran et al. have initially studied the neurodegeneration in the CNS of immunocompetent neonatal C57BL/6, with the results that such an immunocompetent mice can also have susceptibility to ZIKV [35]. BALB/c, which is widely used to prepare the monoclonal antibody, is broadly used in immunological research, especially in vaccine testing of hepatitis C virus (HCV) [36], severe acute respiratory syndrome-associated coronavirus (SARS-CoV) [37], Middle East Respiratory Syndrome coronavirus (MERS-CoV) [38], and so on. Furthermore, as a common mouse model of respiratory syncytial virus (RSV) [39], Japanese encephalitis virus (JEV) [40], and influenza virus [41], BALB/c is believed to be a feasible animal model for ZIKV infection. 

Although all of the strains above are immunocompetent mice, even at their birth, their applicability as new models of ZIKV infection is worth exploring. Semple et al. presented that the stage of CNS development of new born mice was equal to that of a human mid-term fetus [42]. Neonatal rodents were found to have strong susceptibility to Chikungunya virus [43], Arenavirus [44], Borna disease virus (BDV) [45], etc. Rossi et al. [17] connected the infection efficiency with the growth process of mice, and showed that juvenile mice are more susceptible to ZIKV. Recently, an immunocompetent neonatal C57BL/6 mouse model for ZIKV infection was initially proposed [35]. We have reasons to believe that choosing suckling immunocompetent mice as a potential ZIKV infection animal model has infinite potential. Instead, interferon (IFN) deficient models do have their superiority with more severe clinical manifestations. However, the profound immunological defects in these strains do confuse us with question regarding how much of a difference in IFN response will make in the natural pathogenic mechanism of ZIKV. Considering that pathogenicity of *Flavivirus* may be decided not just by the effect of the virus but by the immune response it triggers [46], and an immunocompetent mouse model is urgently needed in order to discover the pathogenesis of ZIKV. Thus, we developed an inventive and normative infective method of ZIKV, i.e., multi-subcutaneous infection. After injecting ZIKV multi-subcutaneously into the backs of suckling mice, we observed signs and symptoms, organ distributions, and pathological changes to determine the application of suckling mouse models to ZIKV infection research. These suckling mouse models represent an important step toward understanding severe neurological outcomes and may be useful for drug screening, vaccine tests, and general ZIKV research. Additionally, by avoiding confounding factors, like transplacental infection and consequent placental insufficiency in brain development, such a neonatal mouse model will contribute to enhancing the understandings about the clinical outcomes of those who are infected in late pregnancy or early childhood.

## 2. Materials and Methods 

### 2.1. Virus

The Asian lineage Z16006 (GenBank no. KU955589.1) was obtained from the Institute of Microbiology in the Center for Disease Control and Prevention of Guangdong Province, China. It was isolated on 16 February 2016 in China from the serum of a patient who travelled to Fiji and Samoa where it happened to be the epidemic country of ZIKV, and its related articles were published in Chinese journals [47,48]. ZIKV strain Z16006 with a single round of amplification on mouse brain cells and three rounds of amplification on C6/36 cells, was grown to 5 × 10^6^ TCID50/mL (50% tissue culture infective dose).

### 2.2. Animals

KM, BALB/c, and C57BL/6 suckling mice (one day old) were purchased from the Animal Experimental Centre of Southern Medical University, Guangdong Province, China. Mice were breast-fed by their own mothers and divided into groups of 5–6 for BALB/c and C57BL/6 and 10–12 for KM. Weights of each nest of mice were measured at birth, and the differences between nests were not statistically significant (*p* > 0.05). The study design was approved by Laboratory Animal Ethics Committee of Southern Medical University (approval number: 2012-041) and animal care was in accordance with institutional guidelines.

### 2.3. Athogenicity Studies

At the age of one day, 20 μL of the inoculum was administered to each mouse strain by multipoint subcutaneous injection (multi-subcutaneous injection) which meant that each suckling mouse was injected with 10^5^ TCID50 ZIKV. Mice of the control group were injected at the same sites with equal amounts of phosphate-buffered saline (PBS). Five inoculation positions were established, including the back of the neck, the centre of the back, the front of the tail, and the waist on both sides, and 4 μL was injected into each site. Post-inoculation, all mice were monitored at least once daily for symptoms and signs of illness (lower limb weakness, upper limb weakness, slow movements, tremble, hunched posture, toe-walking, and neurological manifestations, such as tremors, abnormal gait, paralysis, and circle). Weights were also recorded daily. Organs of mice that were killed to determine viral loads were washed with PBS and stored at −80 °C, and organs of mice that were killed for histopathological examinations were fixed for 16–24 h in 10% paraformaldehyde and stored at 4 °C. During observation, mice that did not respond to stimuli or lost more than 20% of their initial weight (consistent with the Institutional Animal Care and Use Committee (IACUC) protocol) were regarded as moribund and were humanely euthanized by inhaling 40% carbon dioxide (CO_2_). 

### 2.4. Real-Time RT-PCR Assay

Following infection with 20 μL of the inoculum, various organs were harvested, including the brains, livers, spleens, kidneys, and testes, during the clinical course. Blood was collected every day post-infection for viremia detection. Organ samples were washed with PBS thrice, and collected in 1.5-mL Eppendorf tubes. Tubes were weighed and organ weights were determined by subtracting the tube weight. After the organ was ground into homogenate by an electric blender, viral RNA was extracted from 140 μL of tissue homogenate using the QIAamp^®^ (Qiagen, Hilden, Germany) Viral RNA Mini Kit and was quantified by real-time RT-PCR using the LightCycler480^®^ Instrument (Roche Diagnostics, Roche Instrument Center AG, Rotkreuz, Switzerland). Primers ZIKV/F (5′-CVGACATGGCTTCGGACAGY-3′), ZIKV/R (5′-CCCARCCTCTGTCCACYAAYG-3′) and probe (5′-FAM-AGGTGAAGCCTACCTTGACAAGCARTCA-BHQ1-3′) were designed in our own laboratory. 

### 2.5. Histology

At the time of autopsy, the brains, spleens, livers, kidneys, testes, and periocular tissues were harvested and immediately fixed for 16–24 h in 10% neutral buffered formalin. Mice in the control groups were subjected to double-blind pathological diagnosis. Tissues for paraffin embedding were submitted to Guangzhou Huayin Medical Science Company Limited (Guangzhou, China), where they were processed and sectioned before staining with haematoxylin and eosin (H and E) and examined microscopically for histopathological changes. 

### 2.6. Statistical Analysis

Averages of daily weight data were analysed by independent sample *t*-tests with α = 0.05 each day and each group. 

## 3. Results

### 3.1. Infection for ZIKV

Two identical groups of one-day-old KM (*n* = 25), BALB/c (*n* = 25), and C57BL/6 mice (*n* = 25) were multi-subcutaneously inoculated with 20 μL of the inoculant in the back while each control group (*n* = 10) were injected at the same site with equal amounts of PBS. Post-infection symptoms and signs, weight changes throughout disease progression were monitored and compared among the three strains in one group, with another for analysing organ viral distribution, and tissue damage. The experiment was repeated twice using exactly the same process above to test experimental reproducibility. Finally, the data obtained from the two parallel experiments were combined for a unified analysis. 

### 3.2. Clinical Manifestation

According to the statistic of two batches of data, KM (*n* = 50), BALB/c (*n* = 50), and C57BL/6 mice (*n* = 50) were monitored post-infection for symptoms and signs throughout the disease progression. For the three strains, tables of disease progression records were presented (Tables S1 and S3), every single mouse had its own table. Neurological manifestations of each strain were summarized to determine the state of clinical course. From injection to appearance of neurologic symptoms, this period was regarded as incubation which divided into an asymptomatic incubation period and an incubation period with common symptoms without neurologic symptoms. When neurologic symptoms arose, onset was confirmed. For KM and BALB/c suckling mice, the absence of neurological manifestations meant the recovery period. When it came to C57BL/6, with more serious neurological symptoms, they were exacerbated. 

**Figure 1:**
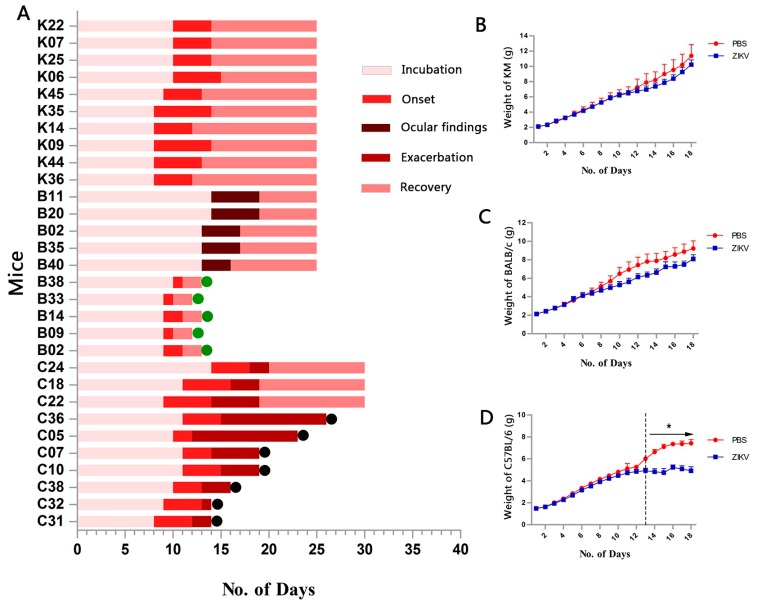
Disease progression and weight changes. (**A**) Disease progression in Kunming mice (KM), BALB/c, and C57BL/6 mice (10 mice per strain). Labels starting with “K” indicate KM, “B” indicate BALB/c, and “C” indicate C57BL/6. The end points were day 25 for KM and BALB/c mice and day 30 for C57BL/6 mice. Among BALB/c, B38, B33, B14, B09, and B02 (*n* = 5) were not sick during their general onset. Green dots show mice used for histopathological examination (*n* = 4). For C57BL/6, black dots show mice that died within 24 h (*n* = 7). (**B**–**D**) Weight changes of each strain were recorded, panel B for KM: phosphate-buffered saline (PBS) (*n* = 6) vs. Zika Virus (ZIKV) (*n* = 17), panel C for BALB/c: PBS (*n* = 5) vs. ZIKV (*n* = 19), and panel D for C57BL/6: PBS (*n* = 6) vs. ZIKV (*n* = 12). One endangered or dead mouse for the C57BL/6 strain was observed on day 11, one on day 13, and two on day 15. Daily weight data were analysed by *t*-tests. * *p* < 0.05.

**Figure 2:**
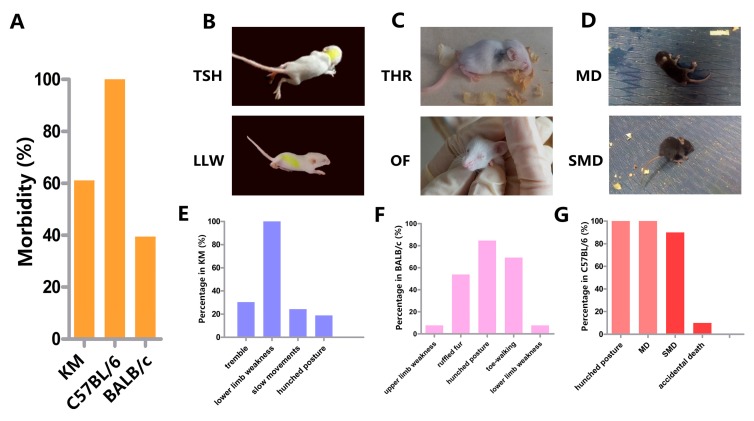
Morbidity, clinical manifestation, and symptom distribution. (**A**) Morbidity. KM (*n =* 50, 62%); BALB/c (*n* = 50, 36%); C57BL/6 (*n =* 50, 100%). (**B**) Clinical manifestation of ZIKV-infected KM, including trembles, slow movement, and hunched posture (TSH) on the upper panel, and lower limb weakness (LLW) on the lower panel. (**C**) Clinical manifestation of ZIKV-infected BALB/c. Toe-walking, hunched posture, and ruffled fur (THR) during onset (upper panel) and ocular findings (OF) detected in remaining mice that were not sick during their general onset (lower panel). (**D**) Clinical manifestation of ZIKV-infected C57BL/6. Movement disorders (MD) during onset (upper panel) and serious movement disorders (SMD) during exacerbation (lower panel). (**E**–**G**) Symptom distribution after ZIKV infection. KM (*n =* 31); BALB/c (*n =* 18); C57BL/6 (*n =* 50). Among C57BL/6, the light red bars indicate onset and the red bars indicate exacerbation. During exacerbation, two mice died accidentally, and all remaining invalid mice manifested SMD.

KM and BALB/c mice exhibited the same disease progression, including incubation, onset, and recovery, but KM mice tended to be more susceptible to challenges with ZIKV based on the distinct neurological clinical manifestation. After incubation (8–10 days), onset (4–6 days) was characterized by slow movement, lower limb weakness, hunched posture, trembling, etc. (Figure 1A and Figure 2B). Lower limb weakness was the only common symptom shared by all sick KM (Movie S1); in these cases, the lower limb was unable to support the body to maintain balance and mice were unable to walk straight. This phenotype was used as an index of onset (Figure 2E) and had a total incidence of 62% (Figure 2A). By day 9 or 10, BALB/c suddenly showed upper or lower limb weakness, hunched posture, ruffled fur, toe-walking, etc. (Figure 2C) and, after a two-day struggle, rapidly recovered, implying a very short onset time (Figure 1A). The symptoms were irregular, and hunched posture and toe-walking had particularly high frequencies, 83.3% and 66.7%, respectively (Figure 2F) (Movie S2). The incidence in BALB/c was 36% (Figure 2A). Interestingly, a slight yellow extraocular exudate was detected in remaining mice by day 13 or 14, and continued for 3–5 days in 55.6% of cases (Figure 2C). The control animals grew normally throughout the trial.

Infected C57BL/6 demonstrated remarkable neurological manifestations and a uniform disease course, unlike KM and BALB/c mice. Disease progression was divided into incubation, onset, exacerbation, and recovery (Figure 1A). Generally, C57BL/6 began to sicken from days 8 to 11, with hunched posture and normal movement, but were unable to hold a balanced position at rest, often swayed, and finally fell. These phenotypes were referred to as movement disorders (MD) (Movie S3 and S4) and led to the highest morbidity, 100% (Figure 2A,D). After 3–5 days, all mice entered the exacerbation stage; they maintained a seriously hunched posture, tiptoe standing position, jumped while walking, easily fell, and were difficult to turn over. We referred to these phenotypes as serious movement disorders (SMD) (Figure 2D,G, Movie S5). The symptoms of most of the mice (37/45) visibly worsened; mice lost movement abilities, struggled to stand, consistently fell, and finally maintained a side-lying position. They commonly died within 24 h and were described as “endangered mice.” Remaining eight mice who were able to stand during exacerbation finally tended to recover, with a single symptom remaining, i.e., a hunched posture. 

All mice gained weight steadily over the study duration, except for C57BL/6 mice. The weights of infected C57BL/6 and their control group were significantly different (*p* < 0.05) beginning on day 13, which coincided with exacerbation. In contrast, KM and BALB/c did not show obvious differences in weight between infected and control mice (*p* > 0.05) (Figure 1B–D).

### 3.3. Organ Viral Loads and Viremia

**Figure 3:**
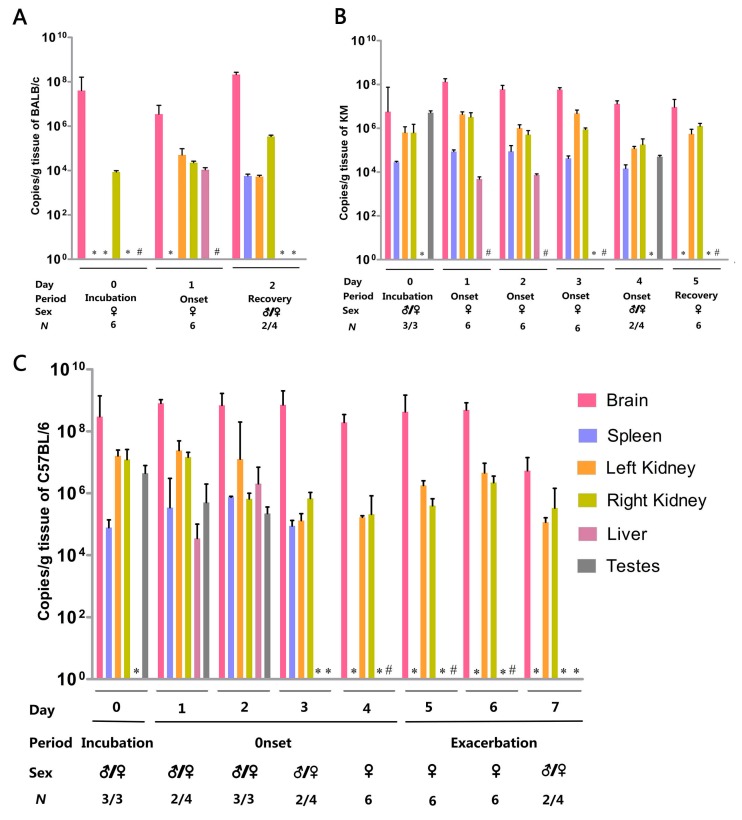
Viral loads during disease progression. (**A**) Viral loads during incubation, onset, and recovery in KM. (**B**) Viral loads during incubation, onset, and recovery in BALB/c. (**C**) Organ titration during incubation, onset, and exacerbation in C57BL/6. Error bars in all panels denote standard errors of the means. # Sample not obtained in female mice; * no detectable titer; ♂ male; ♀ female.

In the process of virus load detection, with the appearance of common symptoms, mice defined as entering the incubation period with common symptoms without neurologic symptoms were euthanized as day 0. Later, each strain was serially euthanized three mice per day during the disease course at onset, recovery, or exacerbation in both repeat experiments, and viral loads were determined in the brains, spleens, livers, kidneys, and the males’ testes. The short error bars of viral loads of six mice from two datasets in each organ show that two experiments obtained a uniform viral distribution and reflected a good repeatability (Figure 3). For all strains, the viral load was highest in the brain. All organs were virus-carrying, but variation was observed among organs.

Only the viral load in the brain of BALB/c mice was high and reached a peak during the recovery period. During the disease course, the virus in the right kidney was detected at all time points, while the left kidney was only virus-infected in the onset and recovery periods. Additionally, the virus was only detected in the liver at the onset period. Only two male animals were sacrificed at the recovery period and their testes were virus-free (Figure 3A).

In KM, the virus was distributed mainly in the brain and kidney. Despite fluctuations, viral loads were consistently maintained at a high level in these two organs. The virus concentration in the brain reached a peak at the first day of onset and then slowly declined. No clear pattern was observed in the kidneys. The virus in the spleen was detected since the incubation period, increased on the first day of the onset period, and dropped to an undetectable level during the recovery period. In the liver, the virus was detected at 1–2 days after the onset of illness. Only three dissected animals at the incubation period and two in day 4 of the onset period were male, and their testes were virus-infected (Figure 3B). 

The virus in the brain and kidney of C57BL/6 was uniformly distributed and was closely related to disease progression. Their virus was observed throughout the entire detection period, demonstrating an M-shaped trend, with apexes at the onset period and exacerbation period and a rapid decline in viral load on day 7. From the incubation period to the onset period, the virus in the spleen initially increased and then decreased, and was entirely purged before the exacerbation period. The virus was only detected during the onset period and was totally eliminated by day 3 in the liver. The testis was virus-infected before day 3 and exhibited a decreasing trend. From day 4–6, all euthanized mice were female, and the testis on day 7 were ZIKV-negative (Figure 3C).

**Figure 4:**
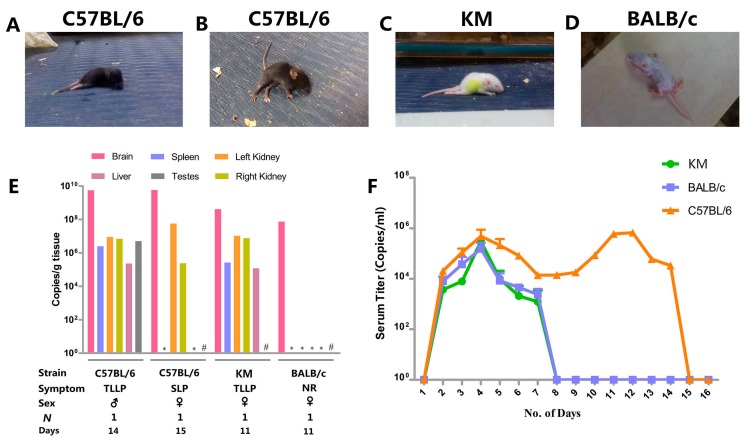
Clinical manifestation and virus distribution for endangered cases and viremia. (**A**–**D**) Clinical manifestation in endangered cases. (**A**) C57BL/6 with TLLP; (**B**) C57BL/6 with SLP; (**C**) KM with TLLP; (**D**) BALB/c with no response (NR); (**E**) Virus distribution of endangered mice. Days: Days post infection. Definitions of “TLLP,” “SLP,” and “NR” are shown in (**A**–**D**). # Sample not taken from female mice, * no detectable titer; (**F**) Viremia. Two mice were analysed per day before day 8, and one mouse was analyzed per day after day 8. The end point for observation was day 16. Error bars in the panel denote standard errors of the means.

There were 36 endangered C57BL/6 mice, including one mouse with two-lower-limb paralysis (Movie S6) and others with side-lying paralysis (Movie S7) (Figure 4A,B). Coincidentally, Kunming mice and BALB/c mice each had one endangered individual, displaying paralysis of two lower limbs (Movie S8) or no response, respectively (Figure 4C,D). C57BL/6 mice with two-lower-limb paralysis carried a high level of virus in each harvested organ, whereas mice with side-lying paralysis only showed signs of the virus in the brain and kidney. Interestingly, another two-lower-limb paralysis KM mouse exhibited large quantities of virus throughout the body, while the BALB/c mouse only carried the virus in the brain (Figure 4E).

An additional 23 suckling mice of each strain were purchased for viremia only once. The blood of each mouse strain was collected once daily post-challenge for viremia detection. Peak plasma viremia occurred on the third day after infection, followed by a slow decline. The virus in the serum of KM and BALB/c mice was eliminated by day 7, while C57BL/6 maintained viremia for a much longer period, with a second peak at day 12, and finally became virus-free at day 15 (Figure 4F). 

### 3.4. Histology

**Figure 5:**
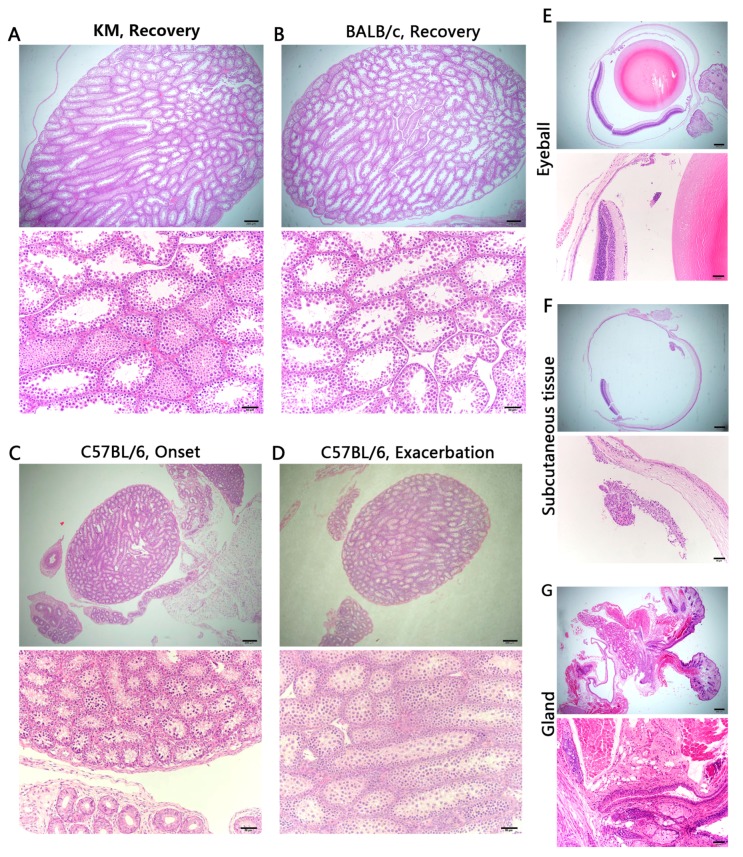
Pathological histology analysis of testis, eyeball, and periocular tissue, hemotoxilyn and eosin (H&E) staining. (**A**–**D**) No overt tissue damage in testes. Sample not obtained in male mice during onset of KM and BALB/c; (**E**–**G**) No unusual findings in eyeball and periocular tissue. Scale bars = 200 μm for upper panels and 50 μm for lower panels in (**A**–**G**).

Organs were harvested at onset and recovery from KM and BALB/c mice, and at onset and exacerbation in C57BL/6 mice with three mice, respectively, in each period in both repeat experiments. Each three BALB/c mice with suspected ocular symptoms were also killed, respectively, at its onset and recovery for eye injuries. Interestingly, the pathological section of two experiments showed a high degree of similarity. Examination of H and E-stained semi-thin sections revealed significant lesions in the brains, spleens, livers, and kidneys, while the testes did not show overt tissue damage associated with ZIKV infection (Figure 5A–D). Additionally, no unusual findings were observed in the periocular tissue of BALB/c mice with suspected ocular symptoms (Figure 5E–G).

**Figure 6:**
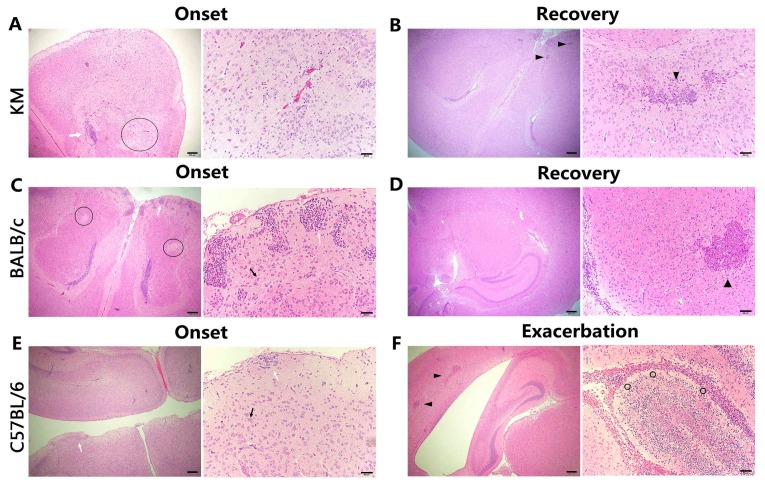
Pathological histology analysis of the brain, H&E staining. (**A**) Left: Encephalomalacia focus (circled) and a glial nodule (white arrow) in the cerebral cortex. Right: Lymphocytic infiltration in the cerebrum tissue. (**B**) Left: Necrosis (arrowheads) was detected, while encephalomalacia focus and glial nodules disappeared in the cerebrum. Right: Necrosis (arrowhead) in the internal granular layer with obvious cellular debris. (**C**) Left: Apparent encephalomalacia focus (circled) in the cerebrum. Right: A glial nodule (white arrow) and neuronal eosinophilic necrosis (black arrow) in the external granular layer. (**D**) Left: Tissue organization in the cerebrum was approximately normal. Right: Necrosis (arrowhead) in the polymorphic layer with an explicit boundary to normal tissue. (**E**) Left: A glial nodule (white arrow) in the molecular layer. Right: A glial nodule (white arrow) and neuronal eosinophilic necrosis (black arrow) in the cerebrum. (**F**) Left: Coagulative necrosis (arrowheads). Right: Overt encephalomalacia focus with an explicit boundary to normal tissue and residual Purkinje cells as ghost cells (circled) in the cerebral cortex. Scale bars = 200 μm for left panels and 50 μm for right panels in (**A**–**F**).

As predicted, the brains demonstrated the most severe pathological changes, showing eosinophilic necrosis, coagulative necrosis, glial cell nodules, ghost cells, neutrophil infiltration, and lymphocytic infiltration. During the onset period in C57BL/6 mice, eosinophilic necrosis of several neurons in the cerebrum and cerebellum was found, and was more severe in the cerebellum, with pieces of coagulative necrosis, while the molecular layer of cerebrum showed apparent glial cell nodules (Figure 6E). Along with exacerbation, residual Purkinje cells as ghost cells appeared, regions of coagulative necrosis in the cerebellum worsened, and encephalomalacia foci in the cerebrum developed (Figure 6F). Moreover, numerous neutrophils were detected in the arachnoid tissue, indicating acute meningitis. Unlike C57BL/6, KM, and BALB/c mice exhibited malacia in the cerebral cortex during onset, when severe glial cell nodules were noticed in the shallow cortex and external granular layer. Additionally, eosinophilic necrosis in BALB/c mice was less serious than that in KM, for which ghost cells were detected. Lymphocytic infiltration in KM suggested the inflammatory response (Figure 6A,C). During recovery, the inflammation disappeared and only necrosis was observed, unlike C57BL/6 mice in the exacerbation stage (Figure 6B,D).

**Figure 7:**
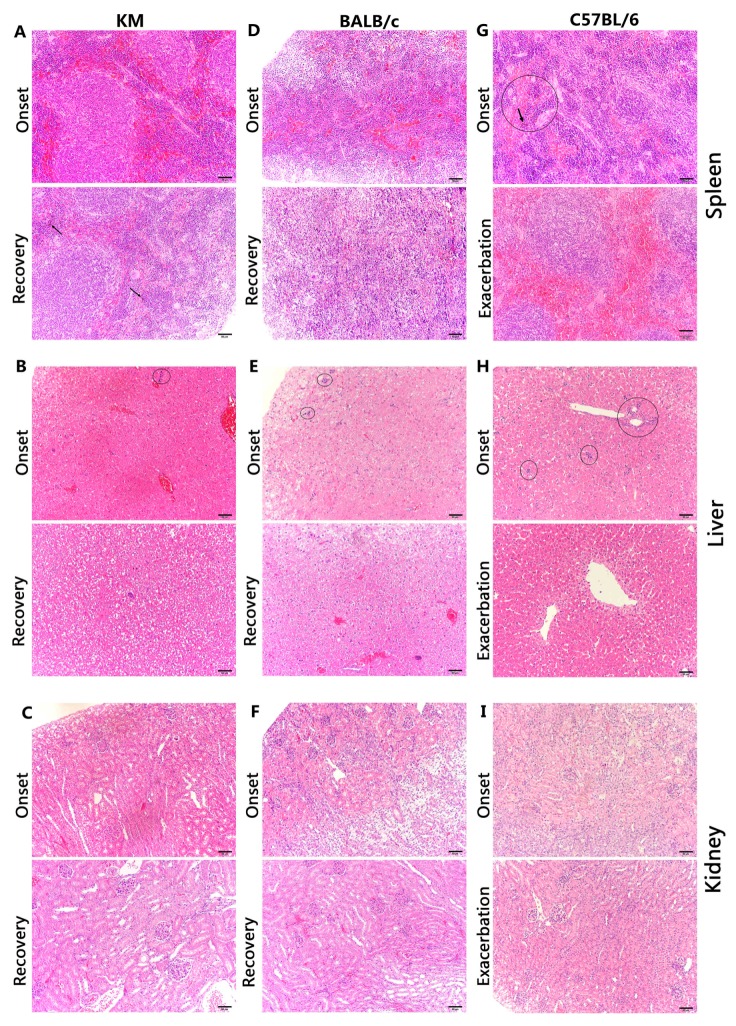
The pathological histology results in the spleen, liver, and kidney. (**A**–**I**) H&Estaining. (**A**–**C**) represent the spleen, liver, and kidney, respectively in KM, BALB/c, and C57BL/6. (**A**) Upper: Hyperaemia in the spleen with reactive hyperplasia of the lymphoid tissue and macrophages. Lower: Hemosiderin (arrows) indicate old haemorrhage. (**B**) Upper: Oedema and hyperplasia of hepatocytes with erythroblastic islands (circled). Lower: Oedema and hyperplasia of hepatocytes. (**C**) Upper: Normal tissue organization in the kidney. Lower: Oedema in the epithelial cells of renal tubules near the renal capsule and hyperaemia in the mesenchyme. (**D**) Upper: Hyperaemia in the spleen with reactive hyperplasia of the lymphoid tissue and macrophages. Lower: Hyperaemia and haemorrhage in the splenic sinusoid with sinus histiocytosis. (**E**) As (**B**). (**F**) As (**D**). (**G**) Upper: Hyperaemia with erythroblastic islands (circled). Hemosiderin (arrows) indicated congestion in the spleen. Lower: Overt hyperaemia with reactive hyperplasia of the lymphoid tissue. (**H**) Upper: Similar to the upper panel in (**B**). Lower: Hepatocyte oedema. (**I**) Normal tissue organization in the kidney. Scale bars = 200 μm for upper panels and 50 μm for lower panels in (**A**–**I**).

During onset in C57BL/6 mice, the splenic sinusoid exhibited remarkable congestion, with a number of erythroblastic islands and macrophages. Additionally, macrophages were detected in splenic tissue, with hemosiderin detected in the cytoplasm, indicating chronic congestion. During exacerbation, erythroblastic islands disappeared, but reactive hyperplasia of the lymphoid tissue and overt germinal tissue emerged (Figure 7G). Interestingly, similar phenotypic effects, like reactive lymphoid hyperplasia in lymphoid follicles of the white pulp, congestion of the splenic sinusoid in the red pulp, and numerous multinucleated giant cells, were found during onset in both KM and BALB/c mice. Both mouse strains demonstrated sinus histiocytosis during recovery, but only KM progressed to old haemorrhagic foci (Figure 7A,D).

In the liver, all three mice exhibited uniform lesions during onset, including hydropic degeneration, renewable hyperplasia, or death of hepatocytes. During recovery, hyperplasia and oedema were noticed in KM and BALB/c (Figure 7B,E). In C57BL/6 mice, liver cell oedema was observed without hyperplasia. Moreover, erythroblastic islands disappeared and hepatocytes near the central venous region were atrophic, indicating chronic congestion (Figure 7H). 

Lesions in the kidney were mild, and all three mice maintained normal structures of nephridial tissue during onset. Oedema was observed in the epithelial cells of the renal tubule near the renal capsule, with hyperaemia in the mesenchyme during recovery in both KM and BALB/c mice, while C57BL/6 maintained normal structures.

## 4. Discussion

A normative multi-subcutaneous dorsal inoculation method was developed to establish suckling mouse models for studies of pathogenic mechanisms, drug screening, and vaccine tests. Mice were injected with 10^6^ TCID50/mL ZIKV in their natural growth state, without genetic deficiency or immune inhibition, to observe the natural pathogenic mechanism of ZIKV infection. The clinical course, manifestation, organ titration, viremia, and histopathology were comprehensively monitored to determine the characteristics of each mouse strain during ZIKV infection. 

The general manifestation and neurological signs and symptoms varied among strains. ZIKV-infected patients not only exhibit common symptoms, but also show occasional neurological manifestations, including GBS and, in particular, microcephaly in infants [49]. Dirlikov et al. [50] analysed 32 cases and summarized the GBS phenotypes associated with ZIKV, such as hyporeflexia or areflexia, leg weakness or paresthesia, arm weakness or paresthesia, and dysphagia. Similar to our results, during onset, C57BL/6 mice were unable to maintain balance, often swayed, and fell, indicating a connection with upper or lower limb weakness. Endangered mice were unresponsive, and this might be related to hyporeflexia or areflexia. In comparison, Manangeeswaran et al. injected suckling C57BL/6 with 2 × 10^3^ PFU ZIKV at day 1 of age, their results had interesting differences with ours. With similar neurological manifestations like unsteady gait, loss of balance and ataxia, their C57BL/6 develop their symptoms around 13 days post infection, and recover two weeks later [35]. Instead, our C57BL/6 have especial exacerbation period after their common onset, which may be due to complex influencing factors. Owing to a lack of diagnostic criteria for GBS in mice, further studies of the relationship between this neurological manifestation and GBS are needed. Moreover, the morbidity reached 100%, supporting the feasibility of C57BL/6 as an animal model for ZIKV infection. Although the morbidity of KM was 61.1%, all invalid mice presented neurological symptoms, such as lower limb weakness. The morbidity of BALB/c mice was only 39.4%, with non-uniform signs and symptoms, but a slight yellow extraocular exudate was detected in some of the remaining unaffected individuals, suggesting the presence of ocular changes. Interestingly, non-purulent conjunctivitis is one of the clinical features [49] of ZIKV infection. Ventura et al. [51] analysed three cases of maculopathy along with microcephaly in children with suspected congenital Zika infection, and de Paula et al. [52] and Miranda et al. [53] reported ocular findings in infants with microcephaly associated with presumed ZIKV congenital infection. Miner et al. [25] found that ZIKV infection in mice causes pan-uveitis with shedding of the virus in tears. Unfortunately, the lack of ocular symptom diagnosis, the difficulty in acquiring excretion and tear samples in suckling mice, and incomplete periocular tissue sections limited our ability to systematically explain the ocular findings in BALB/c mice, and the mechanism should be examined in future studies.

Brains of the three mouse strains which had serious neurological symptoms demonstrated the most severe pathological changes and the highest persistent viral loads. Clinical cases involving foetuses and infants exhibit cerebellar hypoplasia, microglial nodules, and neuronal and glial cell degeneration and necrosis [54], similar to our observations in mice. For example, eosinophilic necrosis of several neurons in the cerebrum and cerebellum was found, and the molecular layer of the cerebrum showed apparent glial cell nodules. Such necrosis and nodules suggest viral replication based on previous findings that ZIKV replicates in both neurons and astroglial cells [55], but this should be confirmed in future studies. Additionally, the cerebellum of C57BL/6 mice had more serious necrosis than the cerebrum. The cerebellum maintains balance, modulates muscle tone, and coordinates voluntary movements, potentially explaining the obvious imbalance and lack of coordination in C57BL/6 mice. Additionally, Purkinje cells as ghost cells were detected in both C57BL/6 and KM mice, but not in BALB/c mice. Purkinje cells are the only neurons that provide outflow and play important roles in motion coordination; they are probably involved in the dyskinesia in C57BL/6 and KM mice. Moreover, lymphocytic infiltration in KM suggested an inflammatory response, similar to the pathological changes reported in clinical cases [54]. Abundant neutrophil infiltration in the arachnoid tissue of C57BL/6 mice indicated acute meningeal infection, in agreement with meningoencephalitis detected in clinical settings [56,57].

Mild pathological changes were observed in the liver and spleen. Early erythroblastic islands exhibited changes in the haematological system, and these may be related to virus dissemination throughout the whole body during long-term viremia. The appearance of chronic ecchymosis in the liver and spleen of C57BL/6 mice revealed high susceptibility. Later, the disappearance of erythroblastic islands coincided exactly with viral clearance, implying a relationship between viral elimination or transfer and the haematological system. Coincidently, reactive lymphoid hyperplasia in lymphoid follicles of the white pulp, induced by humoral immunity by antigenic stimulation of B lymphocytes, was detected in the three mouse strains. 

Pathological changes in the kidney varied among mouse strains. Unlike partial hyperaemia and oedema during recovery in KM and BALB/c mice, C57BL/6 mice maintained normal structures at all time points. However, it is not clear whether high viral loads in the kidneys of KM and C57BL/6 mice suggested that mouse kidneys could persistently carry high concentrations of the virus as a ‘container,’ influencing its own structure. Gourinat et al. [58] found that, compared to serum, the virus could be detected in urine for a longer period of up 10 days and even more than 20 days. In general, through glomerular filtration, tubular resorption, and secretion, urine is derived from blood. Therefore, virus-free serum should correspond to urine without virus. However, after the virus was cleared from the serum, the urine of patients still contained detectable virus, which suggests that ZIKV replication also occurs in human kidneys. Due to the small quantity of urine available from suckling mice, we were not able to determine virus loads in the urine in the present study. However, there is another possibility that biological components in serum which will affect virus replication, different from urine. Additionally, following the kidney, urine will pass the ureters, bladder, and urethra, so we cannot credit the kidney with this discovery. In our study, we did not lucubrate about virus clearance mechanism in serum and urine formation mechanism, so the role that the kidneys play in ZIKV infection still needs further exploration. 

The testes did not show overt pathology, but exhibited detectable viral RNA. Sexual transmission of ZIKV has been observed recently [59]. ZIKV and DENV do not antagonize type I IFN signalling as efficiently in mice compared to humans [60]. Govero et al. [26] treated C57BL/6 mice with a single dose of an anti-IFNAR1-blocking monoclonal antibody, and observed a high viral load and serious pathological changes in the testes. These findings are consistent with those of Lazear et al. [15], who discovered that the testes of *Ifnar1*-/- mice have high viral loads after ZIKV infection. However, it is not clear whether type I IFN is an important regulatory mechanism when ZIKV passes the blood-testis barrier. 

Just like DENV, ZIKV may be restricted in its natural vertebrate host range, generally including primates as its amplification and reservoir hosts [61]. Dudley et al. [24] recently established a rhesus macaque model of Asian-lineage ZIKV infection, which exhibited long-term viremia lasting at least 21 days. Even after apparent clearance of the virus from the blood, viral RNA was detected in the urine and saliva for a certain period, and the virus in the cerebrospinal fluid and plasma was suddenly detected after initial clearance. These results indicate that ZIKV could persist for longer durations in certain tissues at low levels, suggesting its storage in certain parts of the body. Our results indicated that viremia in C57BL/6 mice persists for 14 days, and after apparent clearance of the virus in the serum, ZIKV could persist for longer durations in the brain and spleen, indicating that there might be a ‘container’ for ZIKV. By contrast, viremia in KM and BALB/c mice only continued for seven days. Musso et al. [62] analysed samples obtained from 182 patients, and found that viremia persisted for five days; this was shorter than the duration observed in KM and BALB/c mice. 

In general, major goals for the establishment of animal models are high similarity, feasibility, repeatability, and cost-effectiveness. ZIKV-infected C57BL/6 suckling mice without gene defects or immunosuppression exhibited phenotypes that were most similar to the natural infection status. The highest morbidity (100%), distinctive and easily observable neurological symptoms, regular viral loads in organs, and pathological changes suggested the feasibility and practicality of this animal model. Most of all, with incubation, onset, exacerbation and recovery, our C57BL/6 provide more administration phases and relevant assessment standards for potential therapeutics testing. Additionally, as inbred mice, which benefit from genetic homogeneity and low variation among individuals, C57BL/6 could withstand repeated experiments, and easy to breed at low costs and could be widely applied for drug screening and vaccine tests. By contrast, in spite of lower morbidity, the onset of KM mice exhibited uniform neurological manifestations. With widespread applications in life science research in China, and a long enough prodromal phase for medication around 8–10 days, KM will be an acceptable animal model for Chinese scholars. Despite the low morbidity and non-uniform clinical features, BALB/c mice have unique ocular symptoms which are similar to clinical cases. As a potential useful ZIKV-associated ocular manifestation animal models, BALB/c offers the option of testing conditions that may lead to clinical ocular findings. Despite the application mentioned above, we should also be very cognizant of the limitation that discoveries in such a ZIKV-infected suckling mice model may not be extrapolate to the physiological complications largely found in adults, driving us to conduct further research.

## Supplementary Materials

The following are available online at www.mdpi.com/1999-4915/9/5/165/s1, Table S1: Disease progression record of KM suckling mice: NO. K22 (mouse that recovered), Table S2: Disease progression record of BALB/c suckling mice: No. B14 (mouse without ocular symptoms), Table S3: Disease progression record of BALB/c suckling mice: No. B11 (mouse with ocular symptoms), Table S4: Disease progression record of C57BL/6 suckling mice: No. C36 (mouse that succumbed to infection), Table S5:Disease progression record of C57BL/6 suckling mice: No. C22 (mouse that recovered), Movie S1: Lower limb weakness during onset of KM, Movie S2: Hunched posture and ruffled fur during onset of BALB/c, Movie S3: Movement disorders (MD) during onset of C57BL/6, Movie S4: Movement disorders (MD) during onset of C57BL/6, Movie S5: Serious movement disorders (SMD) during exacerbation of C57BL/6, Movie S6: C57BL/6 with two-lower-limb paralysis (TLLP), Movie S7: C57BL/6 with side-lying paralysis (SLP), Movie S8: KM with two-lower-limb paralysis (TLLP).
