# Supplementary material for: Effective Suckling C57BL/6, Kunming, and BALB/c Mouse Models with Remarkable Neurological Manifestation for Zika Virus Infection
Source: Viruses. 2017 Jun 29;9(7):165. doi: 10.3390/v9070165 (PMC5537657; doi:10.3390/v9070165)
Supplement: Supplementary file 1 [file viruses-09-00165-s001.zip › Table S2.doc]

**Table S2. Disease progression record of BALB/c suckling mice: No. B14 (mouse without ocular symptoms)**

| **Days Post Infection** | **Common Symptoms** | | | | | [**Neurologic**](javascript:void(0);) **Symptoms** | | | | | | **Ocular Symptms** | |
| --- | --- | --- | --- | --- | --- | --- | --- | --- | --- | --- | --- | --- | --- |
|  | **Hunched Posture** | **Ruffled Fur** | **Toe-**  **walking** | **Tremors** | **Slow Movements** | [**Weakness**](javascript:void(0);) [**of**](javascript:void(0);) [**limbs**](javascript:void(0);) | | | | **Movement disorder** | | **Yellow extraocular exudate** | |
|  |  |  |  |  |  | **An Upper Limb** | **Double Upper Limbs** | **A Lower Limb** | **Double Lower Limbs** | **Circle** | **Abnormal Gait** | **Left Eye** | **Right Eye** |
| **1** | **×** | **×** | **×** | **×** | **×** | **×** | **×** | **×** | **×** | **×** | **×** | **×** | **×** |
| **2** | **×** | **×** | **×** | **×** | **×** | **×** | **×** | **×** | **×** | **×** | **×** | **×** | **×** |
| **3** | **×** | **×** | **×** | **×** | **×** | **×** | **×** | **×** | **×** | **×** | **×** | **×** | **×** |
| **4** | **×** | **×** | **×** | **×** | **×** | **×** | **×** | **×** | **×** | **×** | **×** | **×** | **×** |
| **5** | **×** | **×** | **×** | **×** | **×** | **×** | **×** | **×** | **×** | **×** | **×** | **×** | **×** |
| **6** | **×** | **×** | **×** | **×** | **×** | **×** | **×** | **×** | **×** | **×** | **×** | **×** | **×** |
| **7** | **×** | **×** | **×** | **×** | **×** | **×** | **×** | **×** | **×** | **×** | **×** | **×** | **×** |
| **8** | **×** | **×** | **×** | **×** | **×** | **×** | **×** | **×** | **×** | **×** | **×** | **×** | **×** |
| **9** | **√** | **√** | **√** | **×** | **×** | **×** | **×** | **×** | **×** | **×** | **×** | **×** | **×** |
| **10** | **√** | **√** | **√** | **√** | **√** | **√** | **×** | **×** | **×** | **√** | **√** | **×** | **×** |
| **11** | **√** | **×** | **√** | **×** | **√** | **√** | **×** | **×** | **×** | **√** | **√** | **×** | **×** |
| **12** | **√** | **×** | **×** | **×** | **×** | **×** | **×** | **×** | **×** | **×** | **×** | **×** | **×** |
| **13** | **×** | **×** | **×** | **×** | **×** | **×** | **×** | **×** | **×** | **×** | **×** | **×** | **×** |
| **14** | **×** | **×** | **×** | **×** | **×** | **×** | **×** | **×** | **×** | **×** | **×** | **×** | **×** |
| **15** | **×** | **×** | **×** | **×** | **×** | **×** | **×** | **×** | **×** | **×** | **×** | **×** | **×** |
| **16** | **×** | **×** | **×** | **×** | **×** | **×** | **×** | **×** | **×** | **×** | **×** | **×** | **×** |
| **17** | **×** | **×** | **×** | **×** | **×** | **×** | **×** | **×** | **×** | **×** | **×** | **×** | **×** |
| **18** | **×** | **×** | **×** | **×** | **×** | **×** | **×** | **×** | **×** | **×** | **×** | **×** | **×** |
| **19** | **×** | **×** | **×** | **×** | **×** | **×** | **×** | **×** | **×** | **×** | **×** | **×** | **×** |
| **20** | **×** | **×** | **×** | **×** | **×** | **×** | **×** | **×** | **×** | **×** | **×** | **×** | **×** |
| **21** | **×** | **×** | **×** | **×** | **×** | **×** | **×** | **×** | **×** | **×** | **×** | **×** | **×** |
| **22** | **×** | **×** | **×** | **×** | **×** | **×** | **×** | **×** | **×** | **×** | **×** | **×** | **×** |
| **23** | **×** | **×** | **×** | **×** | **×** | **×** | **×** | **×** | **×** | **×** | **×** | **×** | **×** |
| **24** | **×** | **×** | **×** | **×** | **×** | **×** | **×** | **×** | **×** | **×** | **×** | **×** | **×** |
| **25** | **×** | **×** | **×** | **×** | **×** | **×** | **×** | **×** | **×** | **×** | **×** | **×** | **×** |

**× means the symptom was not observed.**

**√ means the symptom was observed.**
