# Supplementary material for: Effective Suckling C57BL/6, Kunming, and BALB/c Mouse Models with Remarkable Neurological Manifestation for Zika Virus Infection
Source: Viruses. 2017 Jun 29;9(7):165. doi: 10.3390/v9070165 (PMC5537657; doi:10.3390/v9070165)
Supplement: Supplementary file 1 [file viruses-09-00165-s001.zip › Table S4.doc]

**Table S4. Disease progression record of C57BL/6 suckling mice: No. C36 (mouse that succumbed to infection)**

| **Days Post Infection** | **Common Symptoms** | | | | | | [**Neurologic**](javascript:void(0);) **Symptoms** | | | | |
| --- | --- | --- | --- | --- | --- | --- | --- | --- | --- | --- | --- |
|  | **Hunched Posture** | **Seriously Hunched Posture** | **Ruffled Fur** | **Toe-**  **Walking** | **Tremors** | **Slow Movements** | **Quiescent State** | | **Motorial State** | | |
|  |  |  |  |  |  |  | **Sway** | **Frequent Drop** | **Short Skips** | **Frequent Drop** | **Tiptoe Standing Position** |
| **1** | **×** | **×** | **×** | **×** | **×** | **×** | **×** | **×** | **×** | **×** | **×** |
| **2** | **×** | **×** | **×** | **×** | **×** | **×** | **×** | **×** | **×** | **×** | **×** |
| **3** | **×** | **×** | **×** | **×** | **×** | **×** | **×** | **×** | **×** | **×** | **×** |
| **4** | **×** | **×** | **×** | **×** | **×** | **×** | **×** | **×** | **×** | **×** | **×** |
| **5** | **×** | **×** | **×** | **×** | **×** | **×** | **×** | **×** | **×** | **×** | **×** |
| **6** | **×** | **×** | **×** | **×** | **×** | **×** | **×** | **×** | **×** | **×** | **×** |
| **7** | **×** | **×** | **×** | **×** | **×** | **×** | **×** | **×** | **×** | **×** | **×** |
| **8** | **×** | **×** | **×** | **×** | **×** | **×** | **×** | **×** | **×** | **×** | **×** |
| **9** | **×** | **×** | **×** | **×** | **×** | **×** | **×** | **×** | **×** | **×** | **×** |
| **10** | **×** | **×** | **×** | **×** | **×** | **×** | **×** | **×** | **×** | **×** | **×** |
| **11** | **√** | **×** | **×** | **√** | **×** | **×** | **√** | **√** | **×** | **×** | **×** |
| **12** | **√** | **×** | **×** | **√** | **√** | **√** | **√** | **√** | **×** | **×** | **×** |
| **13** | **√** | **×** | **√** | **√** | **√** | **√** | **√** | **√** | **×** | **×** | **×** |
| **14** | **√** | **×** | **√** | **√** | **√** | **√** | **√** | **√** | **×** | **×** | **×** |
| **15** | **√** | **×** | **√** | **√** | **√** | **√** | **√** | **√** | **×** | **×** | **×** |
| **16** | **√** | **√** | **√** | **√** | **√** | **√** | **×** | **×** | **√** | **√** | **√** |
| **17** | **√** | **√** | **√** | **√** | **√** | **√** | **×** | **×** | **√** | **√** | **√** |
| **18** | **√** | **√** | **√** | **√** | **√** | **√** | **×** | **×** | **√** | **√** | **√** |
| **19** | **√** | **√** | **√** | **√** | **√** | **√** | **×** | **×** | **√** | **√** | **√** |
| **20** | **√** | **√** | **√** | **√** | **√** | **√** | **×** | **×** | **√** | **√** | **√** |
| **21** | **√** | **√** | **√** | **√** | **√** | **√** | **×** | **×** | **√** | **√** | **√** |
| **22** | **√** | **√** | **√** | **√** | **√** | **√** | **×** | **×** | **√** | **√** | **√** |
| **23** | **√** | **√** | **√** | **√** | **√** | **√** | **×** | **×** | **√** | **√** | **√** |
| **24** | **√** | **√** | **√** | **√** | **√** | **√** | **×** | **×** | **√** | **√** | **√** |
| **25** | **√** | **√** | **√** | **√** | **√** | **√** | **×** | **×** | **√** | **√** | **√** |
| **26** | ***** | ***** | ***** | ***** | ***** | ***** | ***** | ***** | ***** | ***** | ***** |
| **× means the symptom was not observed; √ means the symptom was observed; * means the mouse was endangered.** | | | | | | | | | | | |
